# Dubious presence of *Bartonella bacilliformis* in ticks from Madre de Dios, Peru

**DOI:** 10.1186/s13104-019-4528-1

**Published:** 2019-08-23

**Authors:** Joaquim Ruiz

**Affiliations:** Independent Researcher, P.O.Box 16, 08214 Badia del Valles, Spain

**Keywords:** *Bartonella bacilliformis*, Carrion’s disease, Ticks

## Abstract

*Bartonella bacilliformis* has recently been described in *Amblyomma scalpturatum*, *Amblyomma ovale* and *Rhipicephalus microplus* collected from wild animals in the Peruvian region of Madre de Dios. In this communication, I will discuss the results of a recent study by del Valle-Mendoza et al. together with the *B. bacilliformis* epidemiology. Following my argumentation, I consider the presence of this microorganism in the above ticks improbable.

## Introduction

*Bartonella bacilliformis* is a vector-borne bacteria causing the so-called Carrion’s disease, a biphasic illness which in the absence of treatment can be lethal in up to 88% of patients during its acute phase [1]. This illness currently seems to be restricted to inhabitants from Andean areas of Peru and Ecuador as well as migrants from or visitors to these areas [1]. Additionally, some coastal areas of Ecuador have reported an atypical presentation of the illness [2]. Furthermore, in the mid-1930’s a devastating Carrion’s disease outbreak scourged Southern Colombia resulting in more than 6000 deaths [3]. At present no animal reservoir has been described with asymptomatic carriers to play this role [1]. Indeed *B. bacilliformis* has been detected in the blood of 38% of apparently healthy inhabitants in post-outbreak areas of Carrion’s disease [4].

## Main text

In this context the presence of *B. bacilliformis* has recently been described in three different ticks (*Amblyomma scalpturatum*, *Amblyomma ovale* and *Rhipicephalus microplus*) collected from wild animals in the Peruvian region of Madre de Dios in an area close to the Bolivian border approximately 250 m above sea level [5]. To date, *B. bacilliformis* has only been described as vectorized through sand flies belonging to the genus *Lutzomyia* [1], and only once has the potential of a tick (*Dermacentor andersoni*) been demonstrated to act as a vector in laboratory conditions [6].

The three reported ticks have a wide geographical distribution; *A. scalpturatum* is present in different South American countries including Bolivia, Brazil, Colombia, Ecuador, Guyana, French Guyana, Peru, Suriname and Venezuela, while *A. ovale* may be found from Mexico to Argentina, and *R. microplus* has a cosmopolitan distribution [7–9]. Therefore, the description of new vectors which could expand the disease to new geographical areas might represent a milestone in the study of Carrion’s disease.

Interestingly, from 2013 to 2017, only 2 out of 2276 (0.088%) cases of Carrion’s disease reported in Peru were from Madre de Dios Department [10]. Thus, the apparent ease in detecting *B. bacilliformis* in new potential vectors contrasts with the low number of Carrion’s diseases cases reported in the Department studied. Furthermore, although a recent vector-pathogen adaptation may be suggested, the high number of positives and the wide distribution of these ticks disagrees with the carriage of a severe pathogen such as *B. bacilliformis*, unless transmission of *B. bacilliformis* from these ticks to humans does not occur.

In the commented study [5], the presence of *B. bacilliformis* in tick samples was established by real-time PCR. Nonetheless, while real-time PCR results may be reliable when applied in sterile fluids or on a pure culture, the results obtained by determining the presence or identifying a specific pathogen in microbiota samples should be taken with caution because of the presence of multiple genomes from different microorganisms (with an indefinitely high number undescribed) and (as in this case) that of the host in the sample. Thus, in order to claim the presence of a particular species in a sample such as DNA extracted from a crushed insect, a sufficiently long specific DNA fragment should at least be sequenced. There, it should be mentioned that new *Candidatus* species belonging to the genus *Bartonella* are continually being described [1], including species closely related to *B. bacilliformis* such as *Bartonella ancashensis* (isolated from patients presenting the chronic phase of Carrion’s disease) or *Candidatus Bartonella rondoniensis*, identified in kissing bugs [1, 11]. In most cases, no genome sequence information is available because these species have been detected trough specific PCR in vectors studies or mammal reservoirs [11, 12]. Furthermore, the studies developed to analyze atypical Carrion’s disease in coastal Ecuador areas strongly suggest the presence of undescribed *Bartonella* species [1, 2]. In addition, despite the high real-time PCR specificity when TaqMan probes are used, different reports have shown the possible presence of false positives, even in non-microbiome studies [13]. In this sense, an in silico analysis of the TaqMan probe used in the del Valle-Mendoza et al. study [5] shown that probably might hybridize in other members of the order Rhizobiales, closely related to *Bartonella* spp. In a complex sample such as an insect microbiome, these findings may result in a false positive detection of *B. bacilliformis*, even using a TaqMan probe in real-time PCR assays.

Finally, del Valle-Mendoza et al. [5] reported the use of 55 real-time PCR cycles. On first describing this methodology Li et al. [14] reported a Ct ~ 37 when amplified ~ 1 copy of the selected *B. bacilliformis* target. While we can consider those results showing Ct levels higher than reported minimal Ct as non-quantifying positive, these results most likely belong to unspecific products related to the enormous DNA diversity present in samples or to the presence of an undescribed *Bartonella* spp. having a certain degree of identity in the DNA amplified region. In their study, del Valle-Mendoza et al. [5], do not report either the quantification of *Bartonella* spp. in analyzed samples or the Ct.

In summary, the results obtained by del Valle-Mendoza et al. [5] may reflect the presence of a *Bartonella* spp., perhaps closely related to *B. bacilliformis*. However, following the above discussion, I consider the presence of *B. bacilliformis* highly improbable. In the absence of unequivocal DNA sequencing results, the presence of any specific microorganism in a microbiome sample cannot be claimed.

## Response

By Juana del Valle-Mendoza

Email: jdelvall@upc.edu.pe

Address: Faculty of Health Sciences, Universidad Peruana de Ciencias Aplicadas (UPC), Av. Primavera 2390-Monterrico

**Fig. 1:**
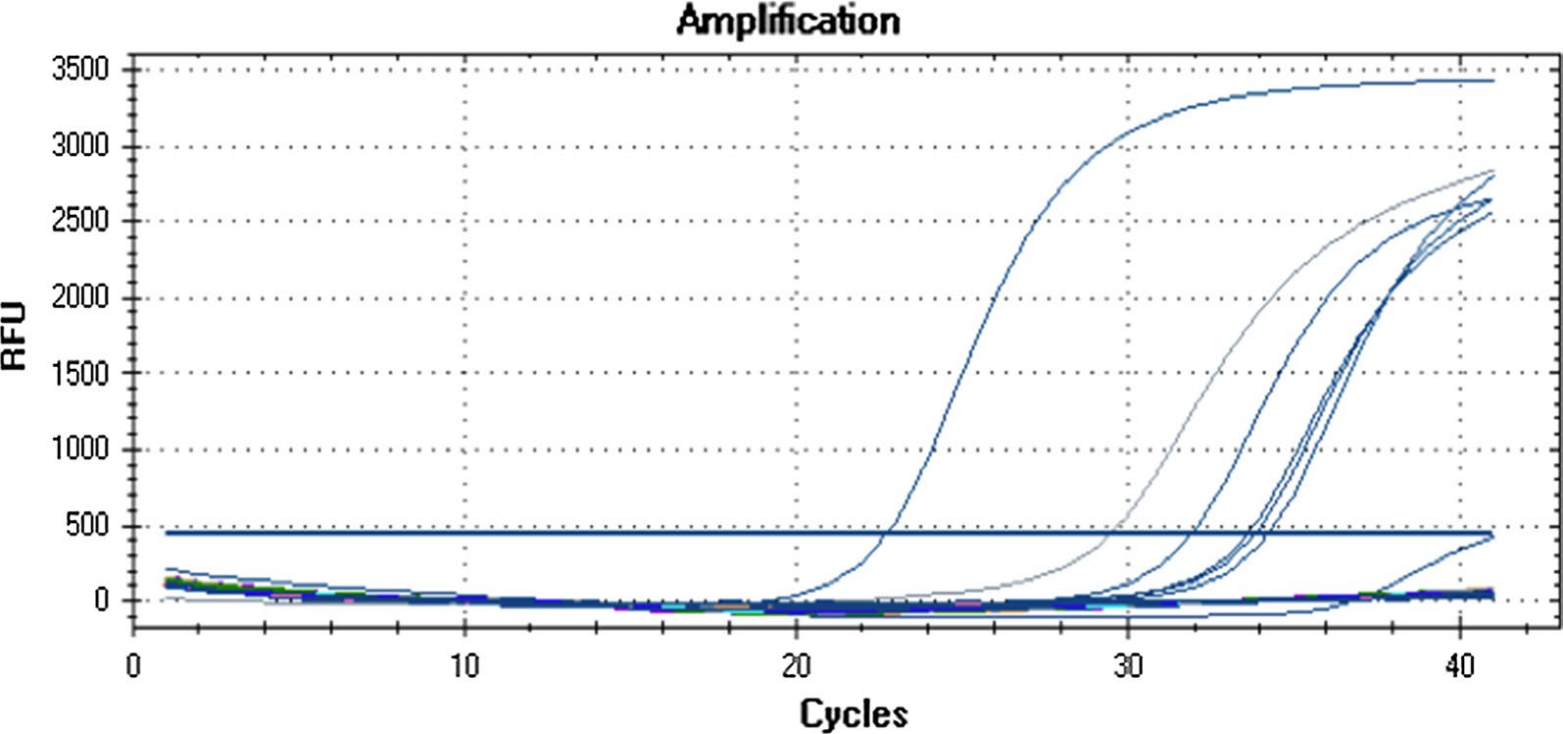
The real time graph for *B. bacilliformis*
  I hope this information rebuts the concerns expressed by Dr. Ruiz in his communication. For us Peruvians, it is crucial to have good diagnosis and we are working for this with the relevant research teams and above all following all the appropriate quality controls.

1. From an epidemiological standpoint, two reported cases were observed between 2013 and 2017 in Madre de Dios; however, there is a concern for bias since both cases were from 2017, where we suspect low-sensitivity diagnostic methods were used.
  Aside for the underreporting of Bartonellosis in Madre De Dios, the use of low-sensitivity diagnostic methods such as thick drop (*gota gruesa*) could explain the low number of cases reported from 2013 to 2017. Additionally, in our study we used PCR real time which has an advantage when compared to the method like the thick drop or conventional PCR which was used during this surveillance period, as it is expressed in the following official document: NTS N° 048 -MINSA/DGSP - V [15].
  This idea is reinforced with our last publication, where *B. bacilliformis* is identified in 30 samples of sera from patients with febrile syndrome in Madre de Dios [16].
  Due to the presence of *B. bacilliformis* in ticks in Madre de Dios and the high prevalence of patients with febrile syndrome, we conducted a second investigation to describe the presence and under-reporting of Bartonellosis in the Peruvian Amazon Basin. Additionally, a study conducted in 2017 reported the presence of Leishmania in ticks [17], suggesting that Bartonella and Leishmania might share the same vector and that Lutzomyia is not the only vector for *B. bacilliformis*.
  The Peruvian government has recognized the need to improve the surveillance of Bartonellosis in Madre de Dios and other affected regions, where there is a high number of undiagnosed patients with vector-related febrile syndrome.
  We are concern that Dr. Ruiz may contradict his previous publication in Spanish titled: “*Enfermedad de Carrión fuera de zonas endémicas. ¿Un riesgo latente?*” [18], in which he stated that the accidental arrival and establishment of a competent vector or the presence of a native arthropod with an intrinsic ability to serve as a vector for *B. bacilliformis* might result in the establishment of native cases in non-endemic regions, given the possibility that these vectors can feed from the blood of asymptomatic carries. Our study also supports this theory proposed by Dr. Ruiz.
  It is not the first time that *B. bacilliformis* was reported in Madre de Dios. In 2004, before the use of molecular techniques, the Peruvian government confirmed an outbreak of Carrión’s disease in seven districts of Madre de Dios using coloration and microscopy techniques. This was stated in our original publication [5] and cited therein as reference 30 and overlooked by Dr. Ruiz. Our work describes a specific event with a specific finding as reported in other scientific publications of the same type, whose primary objective is not to determine the dynamics of the expansion of a disease.
  The fact that a pathogen such as *B. bacilliformis* is present in these ticks and transported by these mammals does not mean that Carrion’s disease expands rapidly. Any disease and particularly those transmitted by vectors, depend on the environment, agent and guest, which makes their transmission and expansion complex.
  The geographic expansion of ticks and isolations of disease-causing bacteria from these is constantly reported [19, 20], *B. bacilliformis* would not be the exception.
2. Dr. Ruiz mentions the geographical distribution of ticks and postulates how the disease can potentially spread and the reasons why it does not happen.
  *Bartonella bacilliformis* is a notifiable disease, however due to the lack of diagnostic resources is not usually made in time and the government uses mainly thick blood smear; although molecular tests are recently being implemented. Initially, our team tried using the PCR for the 16S ribosomal gene, however the primers were not 100% specific, aggregating to the human chromosome 2, thus limiting sequencing. Using real-time PCR has allowed us to improve surveillance and act in coordination with the Regional Health Department.
  The design of the probes was developed by the China CDC team, the results were validated in this laboratory and the amplicons were confirmed by automatic sequencing. The probes and primers, aligned to the blast, are 100% specific for *B. bacilliformis*.
  The identification studies of Bartonella in ectoparasites are becoming more frequent as a Brazilian study by Renan Bressianini do Amaral et al. from 2018 [21] shows.
  In a study from Ulloa et al., in 2018 [22], Dr. Ruiz was an author and actively participated in the preparation of the scientific manuscript. In this work, the same primers, probes and conditions were used for the real-time PCR used in the study under discussion, it was the same technical team and the one who supervised the processing of the samples. We compared the real-time PCR and the conventional PCR of the 16S gene followed by the automatic sequencing, the data can be reviewed in the manuscript [22].
  The primers and probes were designed by the CDC team of China described by Li et al. [23]. The real time graph for *B. bacilliformis* as well as the concentration, Ct of the first and second runs are shown in Table 1 and Fig. 1.

**Table 1:** Concentration and Ct of first and second run

Concentration (ng/μl)	Ct (template volume) 1st test	Ct (template volume) 2nd test (repeat run)
168.9	31.52	31.94
216.6	33.9	33.59
46.4	33.35	33.83
121.1	33	29.46
29	34.33	34.22
113.4	22.97	22.73

### Response

Joaquim Ruiz

Independent Researcher

E-mail: joruiz.trabajo@gmail.com

I have read with enormous interest the answer of the corresponding author of the manuscript “Molecular identification of *Bartonella bacilliformis* in ticks collected from two species of wild mammals in Madre de Dios: Peru” [5] and have the following additional comments.

1. It is well known that the use of PCR is more sensitive than the use of microscopic techniques. Nonetheless, microscopic techniques are performed in all endemic areas, and despite this, *B. bacilliformis* is reported. The acute phase of Carrion’s disease may present severe symptoms, especially if presented in a non-endemic area in which the inhabitants have not acquired immunity. These symptoms may be misinterpreted, but an increase in the number of unexplained severe febrile patients would lead to the subsequent search to determine the causative microorganism. It is true that 18 cases of Carrion’s disease were reported in Madre de Dios in 2004 [24], but a further report published in 2006 classified these cases as not autochthonous [25].
  In no case would the proposed presence of *B. bacilliformis* in *A. scalpturatum*, *A. ovale* and *R. microplus* agree with the risk of the arrival of a competent vector (i.e. some members of the genera *Lutzomyia*) or the presence of a native arthropod which may be competent to carry *B. bacilliformis*. The second option requires that this potential vector should be outside the area of distribution of *B. bacilliformis* (e.g.: first arrival to Carrion’s disease to Colombia in 1934), but these ticks (at least *R. microplus*) are present in different endemic areas. In addition, vector competence for *Leishmania* is not synonymous of vector competence for *Bartonella*.
  These ticks are widely disseminated outside endemic areas, and similarly, one of the mammals studied is widely distributed throughout South America, while the other can be found from Argentina to Southern USA. Indeed, if a pathogen such as *B. bacilliformis* were present in these ticks and (supposedly) carried by these mammals, Carrion’s disease would rapidly expand.
2. Regarding the presence of *B. bacilliformis* in *Lutzomyia maranonensis* the results were fully validated by classical PCR and sequencing which confirm the presence of *B. bacilliformis* [22]. In this study, several samples amplified when RT-PCR was used but not with classical PCR. While a high sensitivity of RT-PCR may be proposed, the presence of other *Bartonellaceae* is also a possible scenario. Indeed, only those *L. maranonensis* pools with confirmed *B. bacilliformis* sequencing were classified as confirmed positives [22]. Identification at a species level in a complex sample such as a crushed insect needs to be confirmed by sequencing or similar tools, especially when a new description is claimed. Sequencing avoids misidentification regarding unspecific annealing or related to the presence of closely related microorganisms such as *B. rondoniensis*. Validation of the procedure in the CDC of China was done in cultured *Bartonella* and not in this type of complex samples. Furthermore, no comparison was made with those *Bartonella* spp. from which no full genome sequence is available, including *B. rondoniensis* which was mentioned above and been proposed to be the microorganisms most phylogenetically close to *B. bacilliformis* [11]. Blast analysis strongly suggests that the probe used may hybridize with other *Rhizobiales*. Taking this into account, it should be highlighted that to date, *B. rondoniensis* has only been described in kissing bugs.
  The correspondence communication I wrote was to comment on a publication [5] which presents a series of data that I consider may be misinterpreted. I state that the most possible scenario is the presence of a microorganism closely related to *B. bacilliformis*. Taking this into account, a closely related undescribed *Bartonella* spp. is causing Peruvian Warts, but not febrile syndromes in coastal Ecuadorian areas [2]. I hope this correspondence piece contributes to a relevant scientific debate because the presence of *B. bacilliformis* in ticks would open the door for dissemination of this pathogen worldwide.

## Data Availability

Data sharing is not applicable to this article as no datasets were generated or analyzed during the current study.
